# Natural Sources and Bioactivities of 2,4-Di-Tert-Butylphenol and Its Analogs

**DOI:** 10.3390/toxins12010035

**Published:** 2020-01-06

**Authors:** Fuqiang Zhao, Ping Wang, Rima D. Lucardi, Zushang Su, Shiyou Li

**Affiliations:** 1 College of Life Science and Bioengineering, Shenyang University, Shenyang 110044, Liaoning, China; zhaofuqiang@iae.ac.cn; 2 CAS Key Laboratory of Forest Ecology and Management, Institute of Applied Ecology, Chinese Academy of Sciences, Shenyang 110016, Liaoning, China; 3 National Center for Pharmaceutical Crops, Arthur Temple College of Forestry and Agriculture, Stephen F. Austin State University, Nacogdoches, TX 75962, USAfuq_zhao@126.com (Z.S.); 4 Southern Research Station, USDA Forest Service, 320 Green Street, Athens, GA 30602, USA; rima.lucardi@usda.gov

**Keywords:** 2,4-di-tert-butylphenol, 2,4-bis(1,1-dimethylethyl)-phenol (2,4-DTBP), 2,4-DTBP, analogs, natural source, bioactivities, autotoxicity, bacteria, fungi, plants, animals

## Abstract

2,4-Di-tert-butylphenol or 2,4-bis(1,1-dimethylethyl)-phenol (2,4-DTBP) is a common toxic secondary metabolite produced by various groups of organisms. The biosources and bioactivities of 2,4-DTBP have been well investigated, but the phenol has not been systematically reviewed. This article provides a comprehensive review of 2,4-DTBP and its analogs with emphasis on natural sources and bioactivities. 2,4-DTBP has been found in at least 169 species of bacteria (16 species, 10 families), fungi (11 species, eight families), diatom (one species, one family), liverwort (one species, one family), pteridiphyta (two species, two families), gymnosperms (four species, one family), dicots (107 species, 58 families), monocots (22 species, eight families), and animals (five species, five families). 2,4-DTBP is often a major component of violate or essential oils and it exhibits potent toxicity against almost all testing organisms, including the producers; however, it is not clear why organisms produce autotoxic 2,4-DTBP and its analogs. The accumulating evidence indicates that the endocidal regulation seems to be the primary function of the phenols in the producing organisms.

## 1. Introduction

2,4-Di-tert-butylphenol or 2,4-bis(1,1-dimethylethyl)-phenol (2,4-DTBP) is a common natural product that exhibits potent toxicity against almost all testing organisms, including the producing species. The phenol has been well investigated in terms of its natural sources and bioactivities, but it has not been systematically reviewed. A basic question has never been addressed: why does an organism produces autotoxic 2,4-DTBP? This review has summarized the available references in both English and Chinese to date. It will provide some basic information to better understand the physiological and evolutionary roles of 2,4-DTBP in the producing organisms.

## 2. Natural Sources

2,4-DTBP is a lipophilic phenol reported in at least 169 species of organisms (see Table 1). 2,4-DTBP was found in 16 species of bacteria in 10 families, such as nitrogen-fixing cyanobacteria [1]; Gram-positive bacteria in hot spring, soils, and food [2,3,4,5,6,7] and Gram-negative bacteria in soil and freshwater [8,9,10,11,12,13]. Some bacteria are causal agents of infectious diseases in humans, e.g., *Microcystis aeruginosa* Kützing, a species of freshwater cyanobacteria that produce neurotoxins and peptide hepatotoxins [12]; and *Vibrio alginolyticus* Miyamoto et al., a marine bacterium causing otitis and wound infection [13]. The phenol has been identified from 11 fungal species of eight families, e.g., edible mushrooms (*Agaricus bisporus* (J.E. Lange) Imbach in Europe and North America and *Lentinus edodes* (Berk.) Pegler in East Asia) [14,15], inedible mushroom (*Trametes suavelens* (L.) Fr.) [16], common mold species in the environment (*Gliomastix murorum* (Corda) S. Hughes, *Aspergillus terreus* Thom, *Didymium iridis* (Ditmar) Fr., and *Penicillium* spp.) [17,18,19,20,21], plant fungal pathogens [22,23], and some prevalent psychrophilic species (*Cryptococcus albidus* (Saito) Skinner) [24].

2,4-DTBP was also reported in different groups of plants, such as diatom *Phaeodactylum tricornutum* Bohlin [25], liverwort *Marchantia polymorpha* L [26], and ferns *Osmunda regalis* L. [27] and *Adiantum venustum* D. Don [28] 2,4-DTBP commonly occurs in the violate or essential oils of many seed plant species. GC-MS analysis showed that 2,4-DTBP occurs in the dichloromethane extracts of the bark via distillation and methanol extracts of the cones and bark of *Pinus yunnanensis* Franch. [29,30], an in *n*-hexane extracts of the cones of *Pinus kesiya* var. *langbianensis* (A. Chev.) Gaussen ex Bui [31]. The analysis also reported that 2,4-DTBP is a major component in the water extracts of fresh needles of *Pinus tabulaeformis* Carr., but not in the fallen leaves or decomposed leaves of the pine [32]. The phenol had a low or non-detectable presence in the rhizosphere soils of a new plantation of Masson’s pine (*Pinus massoniana* Lamb.); however, it became a major compound in the rhizosphere soils of the continuous pine plantation [33].

The phenol is often found in the essential oils of flowering plants, including dicots (107 species, 58 families) and monocots (22 species, eight families) [34,35,36,37,38,39,40,41,42,43,44,45,46,47,48,49,50,51,52,53,54,55,56,57,58,59,60,61,62,63,64,65,66,67,68,69]. In jiangxiang huangtan (*Dalbergia odorifera* T. Chen), it was found that 2,4-DTBP primarily accumulated in the transition tissues between the heartwood and sapwood as the major component in the ethyl acetate extracts (9.64% based on the dry weight) [70]. The concentration of the compound in the slow-growth heartwood is about 0.83% but is not detected in the fast-growth sapwood when using GC-MS [70].

2,4-DTBP has been identified in various animals, such as marine sponge *Zygomycale* sp. of the phylum Porifera [71], centipede *Scolopendra subspinipes* Leach of the phylum Arthropoda [72], spider mite *Tetranychus cinnabarinus* (Boisduval) of the phylum Arthropoda [73], and *Styela clava* Herdman of phylum Chordata [74]. The phenol was also isolated from a praying mantis (*Mantidis ootheca*) egg-case [75].

**Figure 1:**
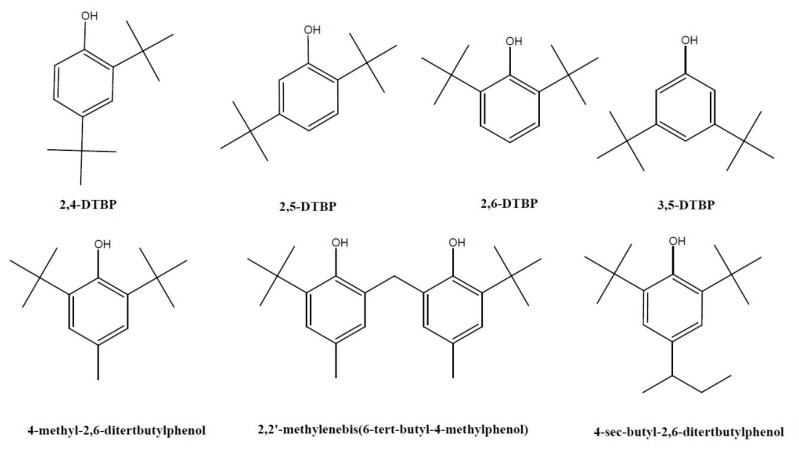
Structures of 2,4-DTBP and its natural analogs.

To date, several natural analogs of 2,4-DTBP have been identified (Figure 1). 2,5-DTBP was found in *Salix* [76], rhizosphere soil of *Boehmeria nivea* (L.) Gaudich. [77], and algal *Grateloupia filicina* C. Ag. [78]. 2,6-DTBP was detected in seeds of *Jastropa curcas* L. [79] and *Metaplexis japonica* (Thunb.) Makino [60]; flowers of *Camellia sasanqua* Thunb. [80], *Aquilaria sinensis* (Lour.) Gilg [45], and *Taxillus chinensis* (DC.) Danser [81]; and leaves of *Chimonanthus* spp. [82]. 3,5-DTBP was reported in flowers of *Aesculus chinensis* [83], fungal *Coriolus versicolor* [84], *Aquilaria sinensis* (Lour.) Gilg [45], whole plants of *Hedyotis lancea* Thunb. [85], and seeds of *Plukenetia volubilis* L. [86]. 4-methyl-2,6-ditertbutylphenol (butylated hydroxytoluene or dibutylhydroxytoluene, BHT) was found in the whole plants of *Praxelis clematidea* (Griseb.) R.M.King & H. Rob. and *Eupatorium catarium* Veldkamp [87], whole plants of *Geum aleppicum* Jacp. [88], and root exudate of sorghum [65]. It is also found in fungal *Nectria* [89]. The lipophilic phenol occurs in some plants, green algae, and cyanobacteria [90,91]. For example, the phenol was reported in rice [69] and *Hedyotis lancea* Thunb. [85]. It was also found in the larval frass of sawyer beetles (*Monochamus alternatus Hope*) [92,93], and female frass of Chinese white pine beetles (*Dendroctonus armandi* Tsai et Li) [94]. It was believed to be produced by the host plant and is concentrated by larvae as a semiochemical compound [93]. However, a later experiment indicated that the phenol was present in the beetle larvae only and not detected in the xylem samples of healthy trees, trees infected with blue-stain fungi, or the wall pupal chambers of *P. massoniana* [95]. 4-sec-butyl-2,6-ditertbutylphenol was found in the stem of *Vernonia amygdalina* Del. [96]. 2,2′-methylenebis(6-tert-butyl-4-methylphenol) was found in the root exudate of sorghum [65]. It is noteworthy that phenols were detected in the sorghum root exudates in the second year of replantation but not in the following years [65].

## 3. Antioxidant Activities

**Figure 2:**
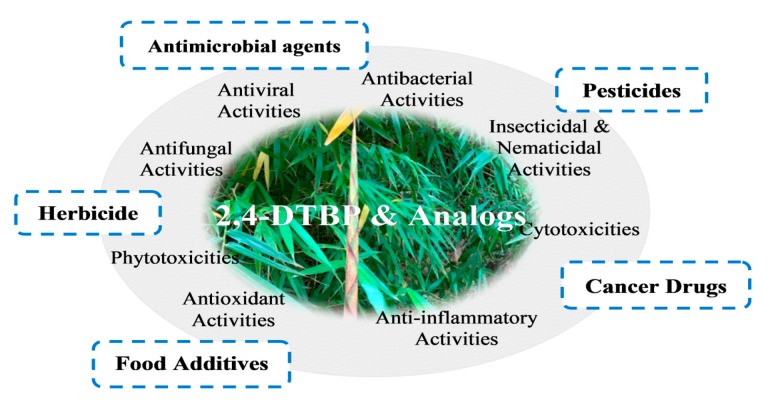
Bioactivities and potential applications of 2,4-DTBP and its natural analogs.

Some investigations on the antioxidant activities of this class of lipophilic phenols were focused on 2,4-DTBP (Figure 2, Table 2). Several in vitro methods for assaying the antioxidant activities have been used, for example, low density lipoprotein (LDL)-oxidation tools, including a thiobarbituric acid reactive substances (TBARS) assay, conjugated diene formation, the relative electrophoretic mobility (REM) of ox-LDL, apoB-100 fragmentation, radical 2,2′-diphenyl-1-picrylhydrazyl (DPPH) scavenging activity, and copper chelating activity, such as in the copper-mediated TBARS assay (IC50: 8.20 mM), 2,2-azobis amidinopropane (AAPH)-mediated oxidation (IC50: 9.9 mM), and 3-morpholino-sydnonimine (SIN-1)-mediated oxidation (29% at 5.0 mM) [72]. 2,4-DTBP from sweet potato extract protects against hydrogen peroxide-induced oxidative stress in the pheochromocytoma cell line (PC12) and in mice [97]. Administration of 2,4-DTBP increased the alternation behavior in mice injected with amyloid-beta peptide (Ab1-42) [97].

The antioxidant activity of BHT was about twice as great as that of 2,4-DTBP because two ter-butyl groups in BHT protect the aromatic hydroxyl group, which forms a phenoxyl radical and donating a hydrogen atom that could quench active free radicals and stop the propagation of lipid peroxidation [98]. The additional ter-butyl group in BHT may also decrease the toxicity. As a result, BHT is one of most commonly used antioxidants for preserving food and feed, and is also listed as an antioxidant food additive by The U.S. Food and Drug Administration (FDA) and the European Union (EU) [99,100]. As an active ingredient from royal jelly, BHT can eliminate 75.86% of ultra-oxygen free radicals at 600 mg/L and 84.47% of the hydroxyl free radicals at 500 mg/L [101]. BHT decreased the Malondiadehyde (MDA) content and increased the superoxide dismutase (SOD) and glutathioneperoxidase (GSH-Px) content in rat liver and serum [101]. The antioxidant activity of BHT can be enhanced in combination use with synthetic 2-ter-butyl-4-methoxyphenol (BHA) and 2,4,6-tri-ter-butylphenl (TBP) [102]. BHT and BHA are fairly heat-stable, [1] but they have been found to exert a dual pro-oxidant and antioxidant action under certain conditions [102]. BHA can stimulate the peroxidase-dependent oxidation of BHT to form the potentially toxic BHT-quinone methide. Among several BHT metabolites, BHT-quinone methide (BHT-QM), 2,6-di-tert-butyl-4-hydroperoxyl-4-methyl-2,5-cyclohexadienone (BHT-OOH), and 3,5-di-tert-butyl-4-hydroxybenzaldehyde (BHT-CHO) have been reported to induce peroxides [102].

## 4. Anti-Inflammatory Activities

Lipopolysaccharide (LPS), the endotoxin found in the cell walls of Gram-negative bacteria, triggers inflammation by activating mononuclear phagocytes (monocytes and macrophages) and results in the production of various pro-inflammatory cytokines. LPS administration was observed to increase the expression of tumor necrosis factor alpha (TNF-α) interleukin *IL-6* and *IL-1b* genes significantly, while 2,4-DTBP treatments were found to decrease the expression of all three genes in the RAW264.7 mouse macrophage cell line [103]. BHT has shown a slight anti-inflammatory activity on the expression of cyclooxygenase-2 (Cox2) and *TNF*-α genes upon stimulation with *Porphyomonas gingivalis* (Pg) fimbriae [102]. The combination of BHT and BHA at a molar ratio of 0.5–2 provides potent anti-inflammatory activity, as tested by gene-expression systems for Cox2 and TNF-α in RAW264.7 cells [102]. The anti-inflammatory activity may be attributable to complex synergistic antioxidant activity [102].

## 5. Cytotoxicities

2,4-DTBP showed a remarkable cytotoxicity against HeLa cells with an IC50 value of 10 μg/mL [6]. 2,4-DTBP exhibited superior effect in the induction of apoptotic genes in cancer cell lines, as did the standard drug Cisplatin [103]. 2,4-DTBP was found to significantly increase the expression of P53 and caspase 7 in both MCF-7 and A431 cell lines, and exhibited significantly higher activation of the P53 gene in MCF-7. Effect of 2,4-DTBP on caspase 7 gene expression was significantly greater in A431, while the effect appeared to be less pronounced in MCF-7 [103].

Based on hepatic and renal toxicity (histopathological changes and an increase in organ weight with blood biochemical changes) in rats, the respective no-observed-adverse-effect levels (NOAELs) for 2,4-DTBP were concluded to be 5 and 20 mg/kg/day [104]. Histologically, there were no obvious changes in uteri and vagina ovariectomized (OVX) CD1 mice between the 2,4-DTBP treatment and the control, and the uterotrophic effect of 2,4-DTBP was not observed in the range of 10 to 250 mg/kg using an oral gavage [105].

It has been reported that long-term and high quantities usage of BHT can induce liver tumors [106]. Due to their pro-oxidant activity, BHT-quinone and BHT-OOH have been reported to result in internucleosomal DNA fragmentation, which is the characteristic of apoptosis [107]. BHT-OOH was found through oxidative DNA damage directly, whereas BHT-quinone was found via DNA damage through H_2_O_2_ generation [107]. After an injection treatment, BHT can considerably increase the number of mitoses in epithelial cell populations from various parts of small intestinal crypts of mice [108]. The effect may be explained by the influence of BHT on the reserve pool of cells and the longevity of individual stages of the mitotic cycle [108]. The BHA/BHT combination (molar ratio 1:1) has inhibited the expression of manganese superoxide dismutase (MnSOD) mRNA in HL60 cells and reversed the transcriptase-polymerase chain reaction (PCR)-activating caspases 3, 8, and 9 [109]. It may contribute to the synergistically antioxidant activity of the BHA/BHT combination and radical-induced formation of intermediates, such as quinone methide [109]. 

## 6. Insecticidal and Nematicidal Activities

2,4-DTBP exhibited significantly adulticidal, larvicidal, ovicidal, repellent, and oviposition-deterrent activities against the spider mite *Tetranychus cinnabarinus* [73]. The mites exhibited the highest run-off rate on bean leaf surfaces sprayed with 2,4-DTBP when applied at sublethal doses and moved toward surfaces that had not been sprayed with the compound, according to Pearson’s v2 test. The compound also showed nematicidal activity against Caenorhabditis elegans during fumigation or soil treatment at temperatures higher than 25 °C [110].

BHT showed larvicidal and ovicidal properties against warehouse beetles (*Trogoderma variabile* Ballion) and black carpet beetles (*Attagenus megatoma* (F.)) [111]. The compound also exhibited lethal insecticidal activity against other beetle species, such as saw-toothed grain beetles (*Oryzaephilus surinamensis* (L.)) and red flour beetles (*Tribolium castaneum* (Herbst)) [112]. The phenol may be used as a preservative in non-toxic aqueous pesticide [113]. It can be used as an adjuvant in a dienol formulation to stabilize p-mentha-1,3-dien-8-ol, an unstable monoterpene alcohol, as a male-produced aggregation-sex pheromone to attract cerambycid beetles (*Paranoplium gracile* (Leconte)) of both sexes in field assays [114]. BHT has been as a component to repel female sawyer beetles [115].

## 7. Antibacterial Activities

Extracellular polymeric substances (EPS) play crucial roles in biofilm formation and biocorrosion, resulting in heavy economic loss in an industrial setup. 2,4-DTBP can modulate the secreted EPS of *Serratia marcescens*, which in turn could facilitate the disruption of biofilms, as well as favoring the diffusion of antimicrobials into the cell aggregates, resulting in the eradication of persistent biofilms [116]. 2,4-DTBP can be used to enhance the efficacy of conventional antibiotics. Intercellular communication in bacteria (quorum sensing (QS)) is an important phenomenon in disease dissemination and pathogenesis that controls biofilm formation. 2,4-DTBP controls QS-mediated biofilm formation and simultaneously increases the hydration of the cell wall, which results in reduced biofilm formation [13].

2,4-DTBP isolated from thermophilic *Bacillus licheniformis* in an Algerian hot spring showed bioactivity against two multidrug resistance bacteria *Pseudomonas aeruginosa* and *Staphylococcus aureus* in pure and mixed cultures that were investigated using a radial diffusion assay at 55 °C [2]. The phenol from *Bacillus*, in association with seaweed, was reported to exhibit a dose-dependent antibiofilm activity against group A *Streptococcus* bacterium [3].

## 8. Antiviral Activity

3-(4,5-Dimethylthiazol-2-yl)-2,5-diphenyltetrazolium (MTT) and plaque reduction assays showed that 2,4-DTBP exhibited significant anti-coxsackievirus B-3 (CVB-3) and anti-herpes virus type 2 (HSV-2) activities [117].

## 9. Antifungal Activities

2,4-DTBP was found to be effective against an agriculturally important root-rot fungus *Fusarium oxysporum* by inhibiting spore germination and hyphal growth [10]. During the fungal spore germination, 2,4-DTBP completely inhibited the germination by preventing the emergence of a normal germ tube and led to the abnormal branching and swelling of hyphae. In such a case, 2,4-DTBP may be binding with *β*-tubulin in microtubules, inhibiting their proliferation and suppressing their dynamic instability as the microtubules are the cytoskeletal polymers in eukaryotic cells and the loss of microtubules should negatively affect the growth rate of spore germination, with an expected reduction in fungal growth in vitro. [10] 2,4-DTBP distinctly reduced the mycelial growth of *Phytophthora capsici* by approximately 50% at 100 µg/mL relative to the control [8]. The germinated seeds of pepper treated with 2,4-DTBP significantly reduced radicle infection by *P. capsici* without radicle growth inhibition [8].

2,4-DTBP had a significant inhibition effect on the mycelium growth at the early stage of culturing tomato leaf mold (*Cladosporium fulvum*) and 0.1 mmol/L of 2,4-DTBP had the best inhibition effect when the mycelium had grown for seven days [118].

The mycelium growth of *Verticillium dahliae* was drastically decreased with increasing concentrations of 2,4-DTBP (0.50 to 2.00 mmol/L) [119].

2,4-DTBP can be produced in some species of *Aspergillus* [18], *Penicillium* [20,21], and *Fusarium* [23], but experiments showed the phenol could inhibit the growth of these fungi. Disc diffusion assays showed that 2,4-DTBP (2 mg/25 mL) prevented the fungal mycelial growth of *Aspergillus niger*, *F. oxysporum*, and *Penicillium chrysogenum* on wheat grains [6]. 2,4-DTBP produced from environmental bacterium *Shewanella algae* strain YM8 significantly reduced the mycelial growth and conidial germination in mold *Aspergillus* [11]. 2,4-DTBP could inhibit *Aspergillus flavus* mycelial growth 7 dpi on potatodextrose agar (PDA) medium at a 5 µg/L concentration and complete inhibition of mycelial growth was observed at 100 µg/L. At 200 µg/L, the compound completely inhibited the germination of conidia. The antimicrobial activity of 2,4-DTBP appeared to correlate with its antioxidative activity because it was able to inhibit the reactive oxygen species (ROS) production in both *Aspergillus* and *Phytophthora cinnamomi* [120]. Thus, the phenol has potential in the development of biopreservatives and dietary antioxidants for food applications.

2,4-DTBP exhibited fungicidal potential at higher concentrations where fluconazole failed to act completely. Various antibiofilm assays and morphological observations revealed that 2,4-DTBP inhibited and disrupted biofilms of *Candida albicans* via the possible inhibition of hyphal development [101]. It also inhibited the production of hemolysins and phospholipases, and secreted aspartyl proteinase, which are the crucial virulence factors required for the invasion of *C. albicans* [121].

## 10. Phytotoxicity: Allelopathy and Autotoxicity

2,4-DTBP shows potential as a natural and environmentally friendly herbicide for weed management [122]. 2,4-DTBP from *Chrysanthemum indicum* inhibited seed germination and seedling growth of lettuce (*Lactuca sativa* var. *ramosa* Hort.), romaine lettuce (*L. sativa* L.), and rapeseed (*Brassica napus* L.) [63].

2,4-DTBP extracted from the rhizome of cogongrass (*Imperata cylindrical* (L.) P. Beauv.) was found to have allelopathic effects on the germination and seedling growth of weedy plants under soilless conditions; for instance, 2,4-DTBP at 0.1 mg/mL showed a 78–95% inhibition of root and shoot growth of beggar ticks (*Bidens pilosa* L.), leucaena (*Leucaena leucocaphala* L. de Wit), and barnyardgrass (*Echinochloa crus-galli* (L.) Beauv) [123]. Lab assays showed that leachates of cogongrass are toxic to ryegrass and lettuce, but not toxic to cogongrass [124]. However, another report showed that boiling water extracts of cogongrass rhizomes that contain catechol, chlorogenic acid, isochlorogenic acid, neochlorogenic acid, p-coumaric acid, p-hydroxybenzaldehyde, scopolin, and scopoletin not only significantly inhibited the seedling growth of five other plant species, but also suppressed cogongrass growth [125]. A later investigation indicated that 2,4-DTBP inhibited 100% of the seed germination and growth of cogongrass at the concentration of 0.1 mg/mL [123].

The phenol also showed toxicity on the root and leaf tissues of the grassy weed *Leptochloa chinensis* (L.) Nees and broadleaf weed *Hedyotis verticillata* (L.) Lam [126] The phytotoxic effect of 2,4-DTBP on these two weeds became apparent at seven days and 14 days after treatment with symptoms of lamina wilting and necrosis, respectively [126]. After a 2,4-DTBP treatment, both had abnormal and much shorter root hairs compared to those of untreated plants. 2,4-DTBP reduced the shoot biomass growth of *L. chinensis* and *H. verticillata* by 50% when applied at concentrations of 50 and 200 µg/mL, respectively [122]. Chuah et al. found that 2,4-DTBP isolated from Napier grass (*Pennisetum purpureum*) exhibited potent herbicidal activity, whereby it completely prevented the root growth of *L. chinensis* in soil at an application rate as low as 0.60 kg a.i. ha^−1^ [127]. 2,4-DTBP induces oxidative stress through the enhanced generation of reactive oxygen species, which cause lipid peroxidation, membrane damage, and the activation of antioxidant enzyme systems, and thus cause a great reduction in chlorophyll content, thereby decreasing chlorophyll fluorescence, transpiration, and the net photosynthetic rate in the leaf tissues [121]. 2,4-DTBP has potent herbicidal properties that can alter the chloroplast ultrastructure, thereby reducing physiological activity of these weedy plants [128]. The present findings imply that 2,4-DTBP may potentially be developed as a soil-applied natural herbicide for the control of *L. chinensis* and perhaps other weeds in an aerobic rice system [127,129].

It was reported that 2,4-DTBP from *P. massoniana* significantly inhibited the seed germination, seed viability, hypocotyl and radicle growth, and seedling growth of Masson’s pine at 0.25–1.0 mg/mL [33]. Another autotoxic study found that 2,4-DTBP had a toxic effect on microorganisms in the rhizosphere soil of hop (*Hamulus lupulus* L.) and affected the photosynthesis and growth of hop seedlings [130,131]. 2,4-DTBP had a significant inhibitory effect on the plant immune system and seed germination of *Atractylodes macrocephala* [132]. 2,4-DTBP from root exudates of chilli pepper showed a medium inhibition against the seed germination and seedling growth of chilli pepper at more than 2 mmol/L [133]. The growth of eggplants was stunted at high concentrations (0.10–1.00 mmol L^−1^) [104]. 2.5-DTBP is one of the compounds responsible for soil sickness in the field of *Boehmeria nivea* [77]. The results of a pot experiment indicated that 2,4-DTBP first significantly decreased and then increased the abundance of culturable bacteria, fungi, and actinomycetes of the rhizosphere soil after treatment [90,91]. 2,4-DTBP from the bulb of *Lilium davidii* var. *willmottiae* and *Fusarium* display a synergetic effect on the *Fusarium* wilt in the lily [134].

## 11. Conclusions

2,4-DTBP is a toxic lipophilic phenol reported in at least 169 species of organisms, such as bacteria (16 species of 10 families), fungi (11 species of eight families), diatom (one species), liverwort (one species), pteridiphyta (two species of two families), gymnosperms (four species of one family), dicots (107 species of 58 families), monocots (22 species of eight families), and animals (five species of five families). To date, several analogs of 2,4-DTBP have been identified in bacteria, algae, fungi, plants, and insects, such as 2,5-DTBP, 2,6-DTBP, 3,5-DTBP, BHT, 4-sec-butyl-2,6-ditertbutylphenol, and 2,2’-methylenebis(6-tert-butyl-4-methylphenol).

The antioxidant and anti-inflammatory activities of 2,4-DTBP have been emphasized in many publications. More importantly, however, the phenol exhibited a broad toxicity in all testing organisms, including the producers; for example, cytotoxicity in human cells and animals, insecticidal and nematicidal activities, antimicrobial activities, and phytotoxicities. However, the available data could not explain why an organism produces such toxic 2,4-DTBP. The endocide theory hypothesizes that an organism is more sensitive to its own endogenous metabolites than external molecules and thus an endocidal compound commonly occurring in different species has a broad spectrum of toxicity or low selective activity [135]. 2,4-DTBP provides a good example. This phenol commonly occurs in diversified organisms and has a potent toxicity against almost all testing organisms.

The following aspects of 2,4-DTBP need to be addressed in future investigations. For example, 2,4-DTBP is usually a major component of volatile oils in many organisms, but its biosynthesis site is not known. A recent report showed that healthy rice plants had level of 2,4-DTBP similar to the plants of the same species following insect herbivory and viral infection [69]; however, a carefully designed experiment is needed to determine whether the production of this phenol can be induced under stresses. Also, the presence of 2,4-DTBP analogs in organisms are often independent of 2,4-DTBP; it is important to elucidate the physiological role of these analogs in the producers. In addition, the bioactivities and potential applications of most analogs of 2,4-DTBP have not been well investigated, although BHT has been commonly used as antioxidants for preserving food and feed.

**Table 1:** Natural sources of 2,4-di-tert-butylphenol (2,4-DTBP).

Family	Biosource	Tissues	Ref.
**Bacteria**			
Bacillaceae	*Bacillus licheniformis*		[2]
	*B. subtilis* Ehrenberg		[3]
Flavobacteriaceae	*Flavobacterium johnsoniae* (Stanier) Bernardet et al.		[8,9]
Microcystaceae	*Microcystis aeruginosa* Kützing		[12]
	*Arthrobacter* sp.		[4]
Nostocaceae	*Nostoc* spp.		[136]
	*Anabaena oryzae* F.E. Fritsch *A. azotica* Ley		[136]
Paenibacillaceae	*Paenibacillus polymyxa* (Prazmowski) Ash et al.		[137]
Pseudomonadaceae	*Pseudomonas monteilii* Elomari et al.		[10]
Shewanellaceae	*Shewanella algae* Simidu et al.		[11]
Streptococcaceae	*Lactococcus* sp.	Cell-free supernatant	[6]
Streptomycetaceae	*Streptomyces globosus* Waksman		[4]
	*S. mutabilis* Pridham et al.		[7]
Vibrionaceae	*Vibrio alginolyticus* Miyamoto et al.	Cell-free culture supernatant	[13]
**Fungi**			
Agaricaceae	*Agaricus bisporus* (J.E. Lange) Imbach		[14]
Bionectriaceae	*Gliomastix murorum* (Corda) S. Hughes		[17]
Glomerellaceae	*Colletotrichum gloeosporioides* (Penz.) Penz. & Sacc.		[22]
Nectriaceae	*Fusarium tricinctum* (Corda) Saccardo		[23]
Omphalotaceae	*Lentinus edodes* (Berk.) Pegler	Caps and stipes	[15]
Polyporaceae	*Trametes suavelens* (L.) Fr.		[16]
Tremellaceae	*Cryptococcus albidus* (Saito) Skinner	Cell-free extract	[24]
Trichocomaceae	*Aspergillus terreus* (Thom)		[18]
	*Didymium iridis* (Ditmar) Fr.		[138]
	*Penicillium flavigenum* Frisvad & Samson	Cells	[20]
	*Penicillium* sp.	Culture	[21]
**Diatom**			
Phaeodactylaceae	*Phaeodactylum tricornutum* Bohlin	Cells	[25]
Liverwort			
Marchantiaceae	*Marchantia polymorpha* L.	Whole thallus	[26]
**Pteridophyta**			
Osmundaceae	*Osmunda regalis* L.		[27]
**Pteridaceae**	*Adiantum venustum* D. Don		[28]
Gyumnasperms			
Pinaceae	*Pinus kesiya* var. *langbianensis* (A.chev.) Gavssen.	Cones	[31]
	*P. massoniana* Lamb.	Rhizosphere soil	[33]
	*P. tabulaeformis* Carr.	Needles	[139]
	*P. yunnanensis* Franch.	Cones and bark	[129,140]
**Dicots**			
Amaryllidaceae	*Allium fistulosum* L.	Root exudates	[141]
Apiaceae	*Anethum graveolens* L.		[142]
	*Centella asiatica* (L.) Urban	Leaves	[143]
Araliaceae	*Panax quinquefolius* L.	Leaves and roots	[144]
Asclepiadaceae	*Metaplexis japonica* (Thunb.) Makino	Seeds	[60]
Asteraceae	*Acroptilon repens* (L.) D.C.	Aerial part	[145]
	*Artemisia annua* L.	Leaves	[34]
	*A. apiacea* Hance		
	*A. japonica* Thunb.		
	*A. capillaris* Thunb.		
	*A. argyi* H.Lév. & Vaniot		
	*A. eriopoda* Bunge		
	*A. tschernieviana* Besser	Aerial parts	[146]
	*Atractylodes coreana* (Nakai) Kitam	Rhizomes	[147]
	*A. macrocephala* Koidz	Rhizomes	[132]
	*Chrysanthemum indicum* L.	Leaves, stem, rot exudates, and rhizosphere soils	[63]
	*Gynura cusimbua* (D. Don) S. Moore	Aerial parts	[148]
	*Xanthium sibiricum* Patr.	Fruits and aerial parts	[149]
Begoniaceae	*Begonia malabarica* Lam.	Fresh plants	[150]
Boraginaceae	*Heliotropium indicum* L.	Aerial parts	[151]
Brassicaceae	*Brassica oleracea* var. *capitata* F. Rubra	Leaves	[152]
	*B. napus* L.	Seeds	[153]
Cactaceae	*Pereskia bleo* (Kunth) de Candolle	Leaves	[154]
Caeselpiniaceae	*Bauhininia variegata* (L.) Benth.	Leaves	[155]
Calycanthaceae	*Chimonanthus* Lindl.		[156]
	*C. praecox* (L.) Link.	Leaves	[82]
	*C. zhejiangensis* M.C. Liu		
	*C. salicifolius* S.Y. Hu		
	*C. nittens* Oliv.		
	*C. grammatus* M.C. Liu		
	*C. campanulatus* R.H.		
Cannabaceae	*Humulus lupulus* L.	Rhizosphere soils	[131]
Capparaceae	*Crateva religiosa* G. Forst.	Stems	[157]
Caprifoliaceae	*Lonicera maackii* (Rupr.) Maxim.	Fruits	[64]
Caricaceae	*Carica papaya* L.	Seeds	[158]
Caryophyllaceae	*Spergularia marina* (L.) Besser	Aerial part	[159]
Combretaceae	*Terminalia travancorensis* Wight & Arn.	Bark	[160]
Convolvulaceae	*Ipomoea batatas* (L.) Lam.	Tubers	[97]
Cornaceae	*Cornus officinalis* Sieb. Et Zucc.	Fruits	[161]
Cucurtibitaceae	*Cucurbita moschata* (Duch. ex Lam.) Duch. ex Poiret	Fruits	[56]
Crassulaceae	*Rhodiola imbricata* Edgew.	Roots	[162]
Equisetaceae	*Equisetum arvense* L.	Whole plant	[163]
Ericaceae	*Rhododendron dauricum* L.	Leaves	[48]
Euphorbiaceae	*Croton bonplandianum* Baill	Leaves	[164]
	*Phyllanthus debilis* Klein ex Willd.	Leaves	[165]
	*Sauropus rostratus* Miq.	Leaves	[55]
Fabaceae	*Albizia julibrissin* Durazz	Leaves and stems	[49]
	*Dalbergia odorifera* T. Chen	Wood	[166]
	*Humboldtia unijuga* Bedd.	Roots	[103]
	*Glycine max* (L.) Merr	Root secretion	[167]
	*Mucuna pruriens* (L.) DC.	Seeds	[168]
	*Vigna radiata* (L.) R. Wilczek	Seeds	[169]
Gentianaceae	*Gentiana apiata* N. E. Br.	Whole plants	[46]
	*G. tibetica* King ex J.D. Hooker	Flowers	[170]
Hydrocharitaceae	*Hydrilla verticillata* (L.f.) Royle	Exudates	[171]
Juglandaceae	*Juglans regia* L.	Root exudates	[172]
Lamiaceae	*Sphenodesme involucrata* var. *paniculata* (C. B. Clarke) Munir	Leaves	[173]
	*Perilla frutescens* (L.) Britton	Leaves	[174]
	*Salvia miltiorrhiza* Bunge	Leaves and roots	[175]
Lauraceae	*Cinnamomum longepaniculatum* (Gamble) N. Chao ex H. W. Li	Leaves	[176]
	*C. loureirii* Nees	Bark	[177]
	*Lindera aggregata* (Sims) Kosterm	Roots	[178]
	*L. angustifolia* (W. C. Cheng) Nakai. *L. rubronervia* (Gamble) Rehder.	Xylem	[179]
	*Persea americana* Mill.	Roots	[120]
Loranthaceae	*Loranthus micranthus* L.	Fresh leaves	[180]
	*L. pentapetalus* Roxb.	Leaves	[181]
	*Viscum ovalifolium* Wallich ex Candolle	Leaves	[181]
Malvaceae	*Cola nitida* (Vent.) Schott & Endl.	Fruits	[182]
Melastomataceae	*Memecylon umbellatum* Burm. f	Leaves	[183]
Menispermaceae	*Tinospora cordifolia* (Willd.) Hook. f. & Thoms.	Embryogenic callus	[184]
Myrtaceae	*Eucalyptus globulus* L.	Leaves	[185]
	*E. grandis* W. Hill ex Maiden	Root	[186]
	*Eugenia dysenterica* D.C.	Fruits	[187]
Nelumbonaceae	*Nelumbo nucifera* Gaertn.	Rhizomes	[188]
Oleaceae	*Olea europaea* L.	Stems	[117]
Paeioniaaceae	*Paeionia lactiflora* Pall.	Root	[189]
Papaveraceae	*Eomecon chionantha* Hance		[67]
Phyllanthaceae	*Phyllanthus emblica* L.	Fruits	[61]
	*Sauropus rostratus* Miq.	Leaves	[55]
Piperaceae	*Piper nigrum* L.	Seeds	[190]
Plumbaginaceae	*Plumbago zeylanica* L.	Roots	[191]
Polygonaceae	*Calligonum polygonoides* L.	Fruits and stems	[192]
	*Polygonum viscosum* Buch-ham	Leaves	[193]
Primulaceae	*Lysimachia foenum-graecum* Hance		[194]
Ranunculaceae	*Aconitum carmichaeli* Dibx.	Root	[68]
	*Clematis connata* D.C.	Whole plant	[195]
	*Consolida regalis* Gray	Stem and leaves	[196]
Rosaceae	*Chaenomeles sinensis* C.K. Schneid.	Fruits	[197]
	*Prunus persica* (L.) Batsch	Roots	[198]
	*Rosa iberica* Stev.	Hips	[199]
	*Sibiraea angustata* (Rehd.) Hand.-Mazz.	Infructescence	[54]
Rubiaceae	*Rubia cordifolia* L.	Stems	[200]
Rutaceae	*Zanthoxylum planispinum* Sieb. et Zucc.	Litters	[201]
	*Nauclea diderrichii* (De Wild. & T. Durand) Merrill	Leaves	[202]
Sapindaceae	*Koelreuteria paniculata* Laxm.	Leaves	[203]
Saururaceae	*Houttuynia cordata* Thunb.	Aerial part	[66]
Scrophulariaceae	*Verbascum phlomoides* L.	Flowers	[204]
Solanaceae	*Capsicum annuum* L.	Root exudates	[133,205]
	*Solanum lycopersicum* var. *cerasiforme* (Dunal) A.Gray	Fruits	[206]
	*S. melongena* L.	Root exudates	[207]
	*Withania coagulans* (Stocks) Dunal	Leaves and micropropagated plant	[208]
Styracaceae	*Sinojackia sarcocarpa* L.Q. Lou	Drupes	[209]
Theaceae	*Camellia sinensis* (L.) Kuntze	Leaves	[210]
Thymelaeaceae	*Aquilaria sinensis* (Loureiro) Sprengel	Resin	[211]
Urticaceae	*Boehmeria nivea* (L.) Gaudich.	Rhizosphere soil	[77]
	*Urtica dioica* L.	Leaves	[212]
Violaceae	*Viola betonicifolia* Sm.	Whole plant	[213]
Vitaceae	*Ampelopsis grossedentata* (Hand.-Mazz.) W.T. Wang		[214]
**Monocots**			
Araceae	*Amorphophallus campanulatus* (Dennst.) Nicolson	Tuber	[215]
Arecaceae	*Cocos nucifera* L. (coconut)	Fruit juice	[216]
Commelinaceae	*Murdannia nudiflora* (L.) Brenan	Whole plant	[62]
Cyperaceae	*Cyperus rotundus* L.	Rhizomes	[217]
	*Heleocharis dulcis* (Burm. f.) Trin.	Rhizomes	[136]
	*Kyllinga triceps* Rottbøll		[218]
Liliaceae	*Lilium davidii* var. *willmottiae* (E.H. Wilson) Raffill	Bulb	[134]
Musaceae	Musa spp.	Root	[219]
Orchidaceae	*Dendrobium moniliforme* (L.) Sw.	Flowers	[220]
	*Gastrodia elata* Blume	Rhizomes	[125]
Palmae	*Phoenix canariensis* Chabaud *Washingtonia filifera* (Lind.) H. Wendl. *Phoenix roebelenii* O’Brien	Leaves	[221]
Poaceae	*Echinochloa crusgalli* (L.) Beauv	Root exudates	[222]
	*Imperata cylindrica* (L.) Beauv	Rhizome and root exudates	[123]
	*Oryza sativa* L.	Root exudate	[223]
	*Pennisetum orientale* Rich.	Aerial part	[47]
	*Pennisetum purpureum* Schumach.	Culm and leaves	[127,129]
	*Phyllostachys pubescens* (Pradelle) Mazel ex J. Houz.	Fresh parenchyma	[224]
	*Sorghum bicolor* (L.) Moench	Root exudate	[65]
	*Spartina cynosuroides* (L.) Roth	Fresh grass	[225]
	*Triticum durum* L.	Seeds	[226]
Zingiberaceae	*Zingiber cassumunar* Roxb.	Rhizomes and leaves	[227]
**Animals**			
Mantidae	*Mantidis ootheca*	Egg cases	[75]
Mycalidae	*Zygomycale* sp.		[71]
Scolopendridae	*Scolopendra subspinipes* Leach	Dried bodies	[72]
Styelidae	*Styela clava* Herdman		[74]
Tetranychidae	*Tetranychus cinnabarinus* (Boisduval)		[73]

**Table 2:** The bioactivities of 2,4-di-tert-butylphenol (2,4-DTBP) and its analogs.

Bioactivities	Chemical Name	Experimental Model	Treatment Doses	Cellular and Molecular Targets	Ref.
Antioxidant Activities	2,4-DTBP	TBARS assay	IC_50_: 8.20 mM	LDL-oxidation	[72]
		Human plasma LDL	IC_50_: 9.9 mM	AAPH-mediated oxidation	[72]
		Human plasma LDL	5.0 mM	SIN-1-mediated oxidation	[72]
		PheochromocytomPC12 cells and mice	2–10 mg/100mL	Hydrogen-peroxide-induced oxidative stress	[97]
		Mice injected with amyloid-beta peptide (Ab1-42)	5–40 mg/kg	Alternation behavior	[97]
	BHT	Ultra-oxygen-free radical	600 mg/L	Radical scavenging	[101]
		Hydroxyl-free radical	500 mg/L	Radical scavenging	[101]
		Liver and serum of rat	100-800 mg/L	MDA, SOD, and GSH-PX content	[101]
Anti-Inflammatory Activities	2,4-DTBP	RAW264.7 mouse macrophage cell line	50 and 100 µg/mL	TNF-α, IL-6, and IL-1b genes	[103]
	BHT	RAW264.7 cells	10 μM	Cox2 and TNF-α genes upon stimulation with Pg	[102]
Cytotoxicities	2,4-DTBP	HeLa cells	IC_50_ value of 10 μg/mL	Cytotoxicity	[6]
		MCF-7 and A431 cell lines	50 and 100 µg/mL	*P53* and caspase 7 generation	[103]
		Rats	5 and 20 mg/kg/day	Respective no-observed-adverse-effect levels (NOAELs)	[104]
		Uteri and vagina ovariectomized (OVX) CD1 mice	10–250 mg/kg by oral treatment	Uterotrophic effect	[105]
	BHT	32P-labeled DNA fragments	50–500 µM	DNA damage	[107]
		Small intestinal crypts of mice		Number of mitoses	[108]
		HL-60 and HSC-2 cells	0.2–0.3 mM	Manganese superoxide dismutase (MnSOD) and reverse transcriptase-polymerase chain reaction (PCR)	[109]
Insecticidal and Nematicidal Activities	2,4-DTBP	Spider mite *Tetranychus cinnabarinus*	LC_50_ values of 1256.51, 625.39, and 743.64 ppm	Adulticidal, larvicidal, ovicidal, repellent, and oviposition-deterrent activities	[73]
		*Caenorhabditis elegans*	0.5–4 g/L	Nematicidal activity	[101]
	BHT	*Trogoderma variabile* Ballion and *Attagenus megatoma* (F.)	0.5 or 2.0%	Larvicidal and ovicidal activity	[111]
		*Oryzaephilus surinamensis* (L.), and *Tribolium castaneum* (Herbst)	10–45 mM	Lethal insecticidal activity	[112]
		A non-toxic aqueous pesticide	1:10 to about 1:600	Preservative treatment	[113]
		*Paranoplium gracile* (Leconte)	5% test solution	Stabilize a male-produced aggregation-sex pheromone	[114]
		Female *Monochamus alternatus*		Repellent activity	[115]
Antibacterial Activities	2,4-DTBP	Biofilm of *Serratia marcescens*	250–300 µg/mL	Secreted etracellular polymeric substances, quorum sensing, and hydration of the cell wall	[13,116]
		*Pseudomonas aeruginosa* and *Staphylococcus aureus* in pure and mixed culture		Antibacterial potency	[2]
		Group A *Streptococcus* bacterium	16–48 µg/mL	Antibiofilm activity	[3]
Antiviral Activity	2,4-DTBP	Coxsackievirus B-3 (CVB-3) and herpes virus type 2 (HSV-2)	6.32 ± 0.67 and 5.24 ± 0.82	Antiviral activity	[117]
Antifungal Activities	2,4-DTBP	Spore and hyphae growth of *Fusarium oxysporum*	1–500 µg/mL	β-tubulin in microtubules	[10]
		*Phytophthora capsici*	100 µg/mL	Mycelial growth	[8]
		Pepper seed infected by *P. capsici*	1–100 g/mL	Radicle infection	[8]
		*Cladosporium fulvum*	0.1 mmol/L	Mycelium growth	[118]
		*Verticillium dahliae*	0.50 to 2.00 mmol/L	Mycelium growth	[119]
		*Aspergillus niger*, *F. oxysporum* and *Penicillium chrysogenum* on wheat grains	2 mg/25 mL	Fungal mycelial growth	[6]
		*Aspergillus*	5–200 µg/L	Mycelial growth and conidial germination ROS production	[11,120]
		Biofilms of *Candida albicans*	2.5–100 µg/mL	Hemolysins, phospholipases, and aspartyl proteinase	[121]
Allelopathy	2,4-DTBP	Seed and seedling of *Lactuca sativa* var. *ramosa* Hort. and *L. sativa* L.	0–0.10 mmol/L	Seed germination and seedling growth	[63]
		Seed and seedling of of *Bidens pilosa* L. and *Leucaena leucocaphala* L. de Wit	0.1 mg/mL	Root and shoot growth	[123]
		Root and leaf tissues of *Leptochloa chinensis* (L.) Nees and *Hedyotis verticillata* (L.) Lam	50 and 200 µg/mL	Lamina wilting and necrosis, and root and shoot growth	[122,126]
		*L. chinensis* in soil	0.60 kg a.i. ha^−1^	Root growth	[127]
		Leaf of weed plant	2.5–100 µg/mL	Reactive oxygen species and chloroplasts	[121,128]
		Seed and seedling *Atractylodes macrocephala*	0.1, 1, and 10 mmol/L	Plant immune system	[132]
		Rhizosphere soil of *Litchi chinensis* Sonn.		Abundance	[90]
Autotoxicity	2,4-DTBP	Seed and seedling of of *Imperata cylindrical* (L.)	0.1 mg/mL	Seed germination and growth	[123]
		Seed and seedling of Masson′s pine	0.25–1.0 mg/mL	Seed germination, seed viability, hypocotyl and radicle growth, and seedling growth	[33]
		Microorganism in the rhizosphere soil of *Hamulus lupulus* L.	7.5 and 15 mmol/m^2^	Photosynthesis and growth of hop seedlings	[130,131]
		Seed and seedling of of *Brassica napus* L., *Echinochloa crus-galli* (L.) Beauv	0.1 mg/mL	Root and shoot growth	[123]
		Seed and seedling of of *Brassica napus* L.	0–0.10 mmol/L	Seed germination and seedling growth	[63]
		Seed and seedling chilli pepper	More than 2 mmol/L	Seed germination and seedling growth	[133]
		Seedling of eggplant	0.10–1.00 mmol/L	Seedling growth	[104]
		Bulb of *Fusarium*		*Fusarium* wilt in the lily	[134]
	2,5-DTBP	*Boehmeria nivea*		Soil sickness in the field	[77]
